# Secondhand smoke and aerosol exposure and awareness among non-smoking workers in a smoke-free workplace: A cross-sectional analysis using urinary cotinine concentrations

**DOI:** 10.18332/tid/226747

**Published:** 2026-10-31

**Authors:** Haruka Miyake, Yohei Inaba, Shota Yoshihara, Takeshi Kochi, Aoi Ito, Shohei Yamamoto, Maki Konishi, Akira Ushiyama, Tetsuya Mizoue

**Affiliations:** 1 Department of Epidemiology and Prevention, Center for Clinical Sciences, Japan Institute for Health Security, Tokyo, Japan; 2 Department of Environmental Health, National Institute of Public Health, Wako, Japan; 3 Department of Health Administration, Furukawa Electric Co., Ltd., Tokyo, Japan

**Keywords:** secondhand smoke, urinary cotinine, smoke-free workplace, occupational health

## Abstract

**INTRODUCTION:**

Although smoke-free workplaces have become increasingly common, residual nicotine exposure among non-smoking workers in such settings remains insufficiently characterized. This study aimed to assess secondhand smoke and aerosol (SSA) exposure and awareness of such exposure among non-smoking workers in a factory with a strict smoke-free workplace policy, using urinary cotinine as an objective biomarker of recent nicotine exposure.

**METHODS:**

As part of an occupational cohort study in Japan, a cross-sectional survey was conducted among employees of a smoke-free factory in April 2024. Participants completed a self-administered questionnaire that included items on smoking habits and SSA exposure and provided first-morning urine samples. Urinary cotinine concentrations were measured and corrected for urinary creatinine. We calculated the prevalence of self-reported SSA exposure and detectable urinary cotinine (≥0.01 ng/mL). Logistic regression was used to examine the association of age, sex, education level, marital status, occupation, shift work, chronic conditions, former smoking, and alcohol use with high urinary exposure (≥0.207 ng/mg creatinine).

**RESULTS:**

Among 746 self-reported non-current smokers, 88.5% had detectable urinary cotinine concentrations, whereas only 21.1% reported SSA exposure from combustible cigarettes or heated tobacco products (HTPs) at least once per month. Higher odds of high urinary cotinine concentrations were observed for female sex (adjusted odds ratio, AOR=1.74; 95% CI: 1.01–2.99), manual work (AOR=1.66; 95% CI: 1.06–2.60), and former smoking (AOR=1.68; 95% CI: 1.18–2.41), whereas higher level of education was associated with lower odds (AOR=0.67; 95% CI: 0.46–0.98). Among 660 participants with detectable urinary cotinine, 78.0% were unaware of their SSA exposure.

**CONCLUSIONS:**

A substantial proportion of non-smoking employees working in a smoke-free factory had detectable urinary cotinine concentrations, whereas only a minority recognized SSA exposure. Further studies are warranted to clarify exposure sources and to better characterize SSA exposure in the context of the increasing use of HTPs.

## INTRODUCTION

Secondhand smoke (SHS) remains a major public health concern because even low-level exposure increases the risks of cardiovascular disease, lung cancer, and respiratory illness among non-smokers^1-3^. With accumulating evidence on the adverse health effects of SHS, smoking restrictions in public places and workplaces have progressively strengthened^4^. However, biomarker studies using cotinine, an established biomarker of environmental tobacco smoke exposure^5^, have suggested that low-level SHS exposure remains common and often unrecognized. Specifically, in the US National Health and Nutrition Examination Survey (NHANES) 2013–2020, 51.2% of non-smoking adults had detectable serum cotinine, and two-thirds of them were unaware of their exposure^6^. In Korean national data between 2009 and 2018, >80% of non-smokers had detectable urinary cotinine despite substantially lower self-reported SHS exposure^7^.

Recognition of SHS exposure may be particularly difficult when exposure is brief or intermittent. This issue may be further complicated in Japan, where heated tobacco products (HTPs) have become widely used. According to the National Health and Nutrition Survey in Japan, approximately 40% of habitual smokers used HTPs in 2024^8^. HTPs emit aerosol rather than combustion smoke and generally generate lower levels of particulate matter and harmful or potentially harmful constituents than combustible cigarettes^9^, making nicotine exposure less perceptible to bystanders.

In Japan, the amended Health Promotion Act, fully implemented in April 2020, made indoor areas of many public facilities, workplaces, and restaurants smoke-free in principle, while allowing designated smoking rooms, including rooms designated for HTP use, under specified conditions^10^. In addition to these legislative measures, workplace tobacco control has been progressively strengthened in Japan, and some companies have implemented complete smoke-free workplace policies^11^. Under such environments, opportunities for nicotine exposure within the workplace are minimized, while exposure outside the workplace may become an increasingly important source of secondhand smoke and aerosol (SSA) exposure among workers. Nevertheless, little is known about the extent to which such exposure remains biologically detectable and whether workers are aware of it. Yet, under complete smoke-free workplace policies, nicotine exposure among workers may increasingly originate from outside the workplace and remain largely unrecognized.

This study aimed to assess SSA and awareness among non-smoking workers in a smoke-free workplace using urinary cotinine concentrations, and to examine background factors associated with high urinary cotinine concentrations.

## METHODS

### Study design and participants

This cross-sectional study was conducted as an ancillary survey of an ongoing multicenter epidemiological study of workers in Japan^12^. In April 2024, a survey was conducted during annual health checkups at a participating company’s smoke-free factory, where the use of all tobacco products, including cigarettes and heated tobacco products, is prohibited in all areas, including outdoors, and workers are not permitted to leave the premises for these purposes during work hours or lunch breaks. Participants were asked to complete a self-administered questionnaire regarding smoking habits, SSA exposure, and occupational and behavioral factors, and to provide urine samples for biomarker assessment. Anthropometric measurements, biochemical data, and medical history were obtained from health checkup records. Workers were included in the present analysis if they underwent the periodic health checkup and agreed to participate in the study. Those with missing information on smoking status or without a urine sample were excluded. For the main analysis among non-smokers, users of any tobacco product were further excluded. The study was conducted in accordance with the Declaration of Helsinki and was approved by the Institutional Ethics Committee (approval number: JIHS-S-001140). Written informed consent was obtained from all participants before data collection.

### Measurement of urinary creatinine and cotinine

Participants were instructed to collect first-morning urine samples in designated collection tubes and submit them at the survey reception desk during the health checkup. Urinary creatinine concentrations were measured using an enzymatic method at a commercial laboratory (LSI Medience Corporation, Tokyo, Japan). The remaining aliquots were transported to the National Institute of Public Health and stored at -80°C until biochemical analysis.

Urinary nicotine metabolites, including cotinine, were measured by liquid chromatography–tandem mass spectrometry (LC-MS/MS), with the addition of an enzymatic hydrolysis step to convert cotinine glucuronide to free cotinine. Briefly, urine samples were incubated for 6 h at 37°C with 1000 units of β-glucuronidase from Helix pomatia (SigmaAldrich, St. Louis, MO, USA; catalog no. G7017) in 100 mM ammonium acetate buffer (pH 4.50). The hydrolyzed samples were then processed and analyzed^13^. Accordingly, cotinine concentrations are reported as total cotinine, comprising free cotinine and cotinine glucuronide. The limit of quantification for urinary cotinine was 0.01 ng/mL. To account for urinary dilution, quantified cotinine concentrations were normalized to urinary creatinine concentrations and expressed as ng/mg creatinine. Participants with cotinine concentrations below the limit of quantification were classified as having undetectable cotinine.

We visually inspected the distribution of urinary cotinine concentrations among all participants, including smokers. The cotinine concentration corresponding to the lowest frequency between the peaks was used as the cutoff separating typical smokers from non-smokers. Among self-reported non-current smokers with detectable cotinine concentrations below this cutoff, participants were further categorized into tertiles according to cotinine concentration. Participants with cotinine concentrations greater than the cutoff were classified as the very high exposure group. Thus, four urinary cotinine categories (Tertile 1 [low], Tertile 2, Tertile 3 [high], and very high exposure) were created among self-reported non-current smokers. Tertile 3 and the very high exposure group were combined and defined as the high exposure group for regression analyses.

### Smoking, SSA exposure, and other variables

Information on demographic, occupational, and behavioral factors was obtained from questionnaires and health checkup records. Participants were asked about current and past smoking habits, including use of combustible cigarettes, HTPs, and other tobacco products. They were also asked about the frequency of SSA exposure separately for combustible cigarettes and HTPs in any settings, including homes, workplaces, restaurants, bars, building entrances, and other public spaces, with response categories ranging from ‘never’ to ‘daily’. Participants who reported exposure to either tobacco type at least once per month were considered to be aware of SSA exposure in daily life. Background factors considered in the analysis were sex, age, education level, marital status, occupation, work shift, chronic condition limiting daily activity, former smoking, and alcohol use.

### Statistical analysis

The proportions of participants with self-reported SSA exposure, detectable urinary cotinine concentrations, and high detectable urinary cotinine concentrations (≥0.207 ng/mg creatinine) among self-reported non-current smokers were calculated and expressed as percentages. These proportions were evaluated according to sex (male, female), age (<40 or ≥40 years), education level (<13 or ≥13 years), marital status (married, other), occupation (non-manual, manual), work schedule (day shift, night/rotating shift), chronic condition limiting daily activity (no, yes), smoking status (never, former), and alcohol use (<1 or ≥1 day/week). Differences between groups were tested using the chi-squared test. Univariable logistic regression analysis was used to estimate crude odds ratio (OR) and 95% confidence interval (CI) for having high detectable nicotine exposure, defined as belonging to the highest tertile group and the very high exposure group combined, in relation to each covariate. We also ran a multivariable logistic regression model including all the abovementioned variables to estimate the adjusted odds ratio (AOR). To evaluate discrepancies between biomarker-based nicotine exposure and self-reported SSA exposure, we calculated the proportion of participants who were aware of SSA exposure according to urinary cotinine category and tested the trend among those with detectable cotinine using the Cochran–Armitage trend test. All analyses were performed using Stata version 18.0 (StataCorp LLC, College Station, TX, USA), and a two-sided p<0.05 was considered statistically significant.

## RESULTS

As shown in the participant flow diagram (Figure 1), of 1322 workers who underwent the periodic health checkup, 1024 agreed to participate in the study. After excluding those who were missing smoking status (n=1) and those who did not provide a urine sample (n=132), data from 891 participants were used to determine urinary cotinine cutoffs distinguishing typical smokers from non-smokers. Of these, we excluded participants who reported current use of combustible cigarettes, HTPs, or other tobacco products, leaving 746 self-reported non-current smokers for the analysis of SSA exposure.

**Figure 1:**
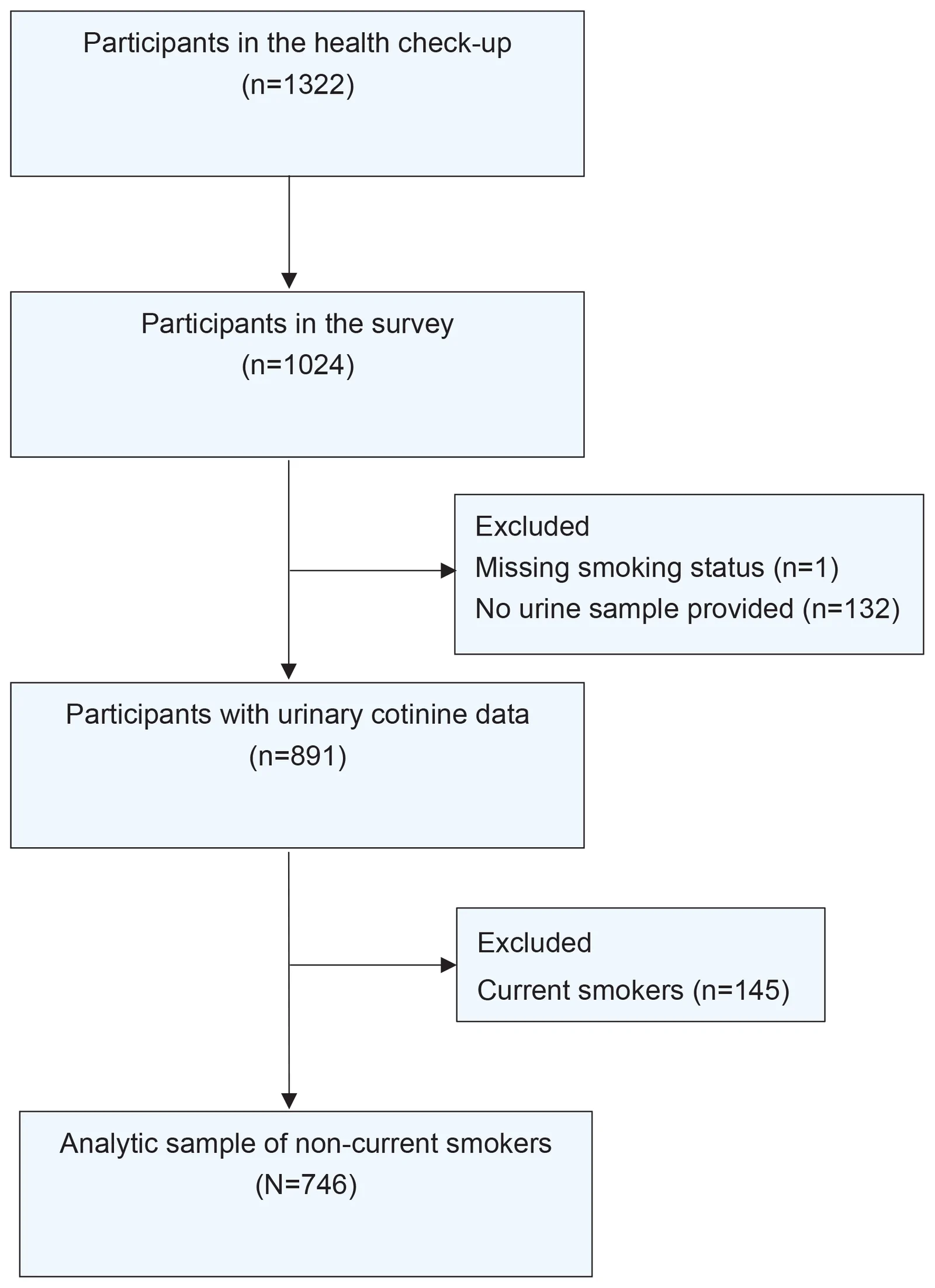
Flow diagram of participant selection among workers in a smoke-free factory, J-ECOH Study

Among all participants, including smokers, creatinine-corrected urinary cotinine concentrations showed a bimodal distribution with two distinct peaks (Figure 2). The nadir between the two peaks was observed at 12.18 ng/mg creatinine.

**Figure 2:**
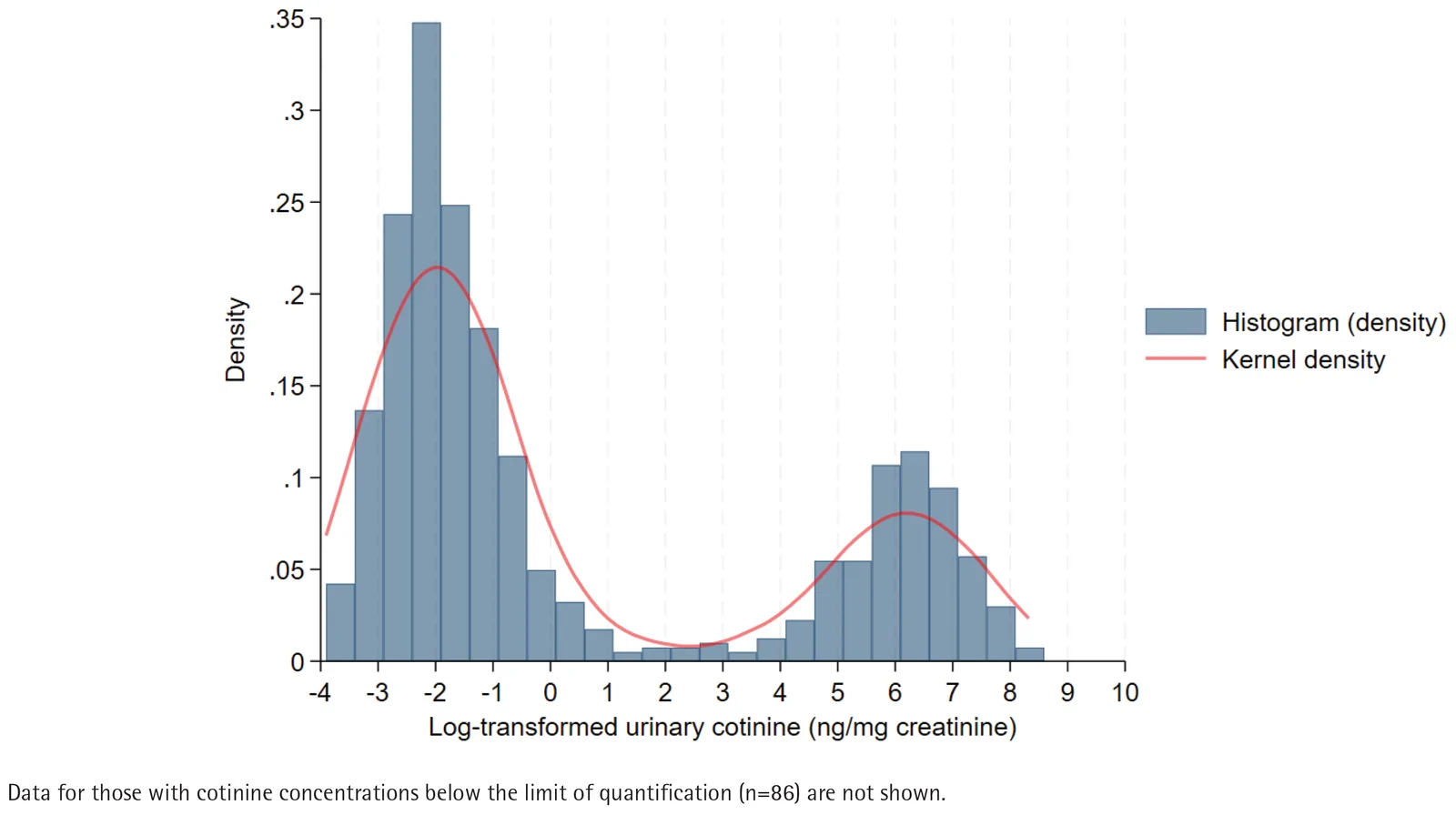
Distribution of creatinine-corrected urinary cotinine concentrations among all participants in a smoke-free factory, J-ECOH Study (N=805)

Among 746 non-smokers, 671 (89.9%) were men, and the mean age was 46.5 years (SD=12.5). As shown in Table 1, 660 participants (88.5%) had detectable urinary cotinine concentrations, whereas only 21.1% reported SSA exposure from either combustible cigarettes or HTPs at least once per month. Women, younger workers, those with lower education level, unmarried workers, manual workers, and shift workers had a higher prevalence of self-reported SSA exposure than their counterparts, although the differences were not statistically significant except for marital status (married 17.7% vs other 28.3%, p<0.01). In contrast, the proportion of those with cotinine-detected SSA exposure was consistently high across background subgroups, exceeding 85% in all subgroups. The only exception was sex, with women showing a significantly higher proportion than men (97.3% vs 87.5%, p<0.01).

**Table 1:** Prevalence of self-reported and cotinine-detected secondhand smoke or aerosol exposure among non-smoking workers in a smoke-free factory, Japan, a cross-sectional analysis of the J-ECOH Study (N=746)

*Characteristics*	*Total* *n*	*Self-reported SSA exposure*		*Cotinine-detected SSA exposure*	
		*Prevalence (%)*	p*	*Prevalence (%)*	p*
**Overall**	746	21.1		88.5	
**Sex**					
Male	671	20.6	0.34	87.5	0.01
Female	75	25.3		97.3	
**Age** (years)					
<40	204	24.5	0.15	88.2	0.90
≥40	542	19.7		88.6	
**Education level** (years)					
<13	370	23.8	0.07	89.7	0.28
≥13	375	18.4		87.2	
**Marital status**					
Married	509	17.7	<0.01	87.6	0.29
Other	237	28.3		90.3	
**Occupation**					
Non-manual	460	18.7	0.06	88.3	0.93
Manual	259	24.7		88.0	
**Work schedule**					
Day shift	607	19.9	0.12	88.3	0.76
Night/rotating shift	139	25.9		89.2	
**Activity-limiting chronic condition**					
No	563	19.7	0.12	88.6	0.81
Yes	183	25.1		88.0	
**Smoking status**					
Never	449	21.4	0.78	89.1	0.52
Former	297	20.5		87.5	
**Alcohol use** (days/week)					
<1	404	22.3	0.37	89.1	0.55
≥1	342	19.6		87.7	

SSA: secondhand smoke and aerosol. Totals may not equal the overall analytic sample because of missing values for some covariates.

* Calculated using the chi-squared test.

In the logistic regression analysis (Table 2), higher adjusted odds of high urinary cotinine concentrations were observed for female sex versus male sex (AOR=1.74; 95% CI: 1.01–2.99), manual work versus non-manual work (AOR=1.66; 95% CI: 1.06–2.60), and former smoking versus never smoking (AOR=1.68; 95% CI: 1.18–2.41), whereas higher level of education versus lower level was associated with lower odds (AOR=0.67; 95% CI: 0.46–0.98). The significant association observed for night or rotating shift work versus day shift in the univariable analysis (OR=1.53; 95% CI: 1.06–2.23) was largely attenuated and no longer statistically significant after multivariable adjustment.

**Table 2:** Associations between high urinary cotinine concentrations and background factors among non-smoking workers in a smoke-free factory, Japan, a cross-sectional analysis of the J-ECOH Study (N=746)

*Variables*	*Total* *n*	*High cotinine exposure^a^*		
		*Prevalence (%)*	*OR (95% CI)*	*AOR (95% CI)*
**Overall**	746	37.7		
**Sex**				
Male (ref.)	671	37.3	1.00	1.00
Female	75	41.3	1.19 (0.73–1.93)	1.74 (1.01–2.99)
**Age** (years)				
<40 (ref.)	204	32.8	1.00	1.00
≥40	542	39.5	1.33 (0.95–1.87)	0.99 (0.64–1.52)
**Education level** (years)				
<13 (ref.)	370	46.2	1.00	1.00
≥13	375	29.3	0.48 (0.36–0.65)	0.67 (0.46–0.98)
**Marital status**				
Married (ref.)	509	36.7	1.00	1.00
Other	237	39.7	1.13 (0.82–1.55)	1.24 (0.85–1.83)
**Occupation**				
Non-manual (ref.)	460	30.7	1.00	1.00
Manual	259	47.1	2.01 (1.47–2.76)	1.66 (1.06–2.60)
**Work schedule**				
Day shift (ref.)	607	35.8	1.00	1.00
Night or rotating shift	139	46.0	1.53 (1.06–2.23)	0.92 (0.56–1.50)
**Activity-limiting chronic condition**				
No (ref.)	563	36.6	1.00	1.00
Yes	183	41.0	1.20 (0.86–1.69)	1.14 (0.79–1.64)
**Smoking status**				
Never (ref.)	449	31.2	1.00	1.00
Former	297	47.5	1.99 (1.47–2.70)	1.68 (1.18–2.41)
**Alcohol use** (days/week)				
<1 (ref.)	404	34.9	1.00	1.00
≥1	342	40.9	1.29 (0.96–1.74)	1.36 (0.97–1.92)

AOR: adjusted odds ratio. CI: confidence interval. The multivariable logistic regression model included all background factors listed in the table; each AOR was adjusted for all other variables in the table. Totals may not equal the overall sample due to missing values for some covariates.

a Urinary cotinine concentrations ≥0.207 ng/mg creatinine.

Of the 660 non-smokers with detectable urinary cotinine concentrations, only 145 (22.0%) were aware of their SSA exposure, while 515 (78.0%) were unaware of such exposure (Table 3). Awareness was lowest in the lowest tertile of urinary cotinine concentration (15.9%), increased to 27.0% in the highest tertile, and decreased to 19.4% in the very high group, with no statistically significant trend across urinary cotinine categories (p for trend=0.14).

**Table 3:** Awareness of secondhand smoke or aerosol exposure according to urinary cotinine category among non-smoking workers in a smoke-free factory, Japan, a cross-sectional analysis of the J-ECOH Study (N=746)

*Self-reported exposure*	*Urinary cotinine (ng/mg creatinine)*					
	*< LOQ ^a^*	*Tertile 1 (<0.1)*	*Tertile 2 (0.10–0.206)*	*Tertile 3 (0.207–9.552)*	*Very high (≥12.18)*	*Cotinine quantified^b^*
**Total**, n	86	189	189	189	93	660
**Not exposed**	74 (86.1)	159 (84.1)	143 (75.7)	138 (73.0)	75 (80.7)	515 (78.0)
**Exposed**	12 (14.0)	30 (15.9)	46 (24.3)	51 (27.0)	18 (19.4)	145 (22.0)

Data are presented as n (%). Percentages may not total 100% because of rounding. P-value for trend was 0.14 among 660 participants with quantified cotinine, calculated using the Cochran–Armitage trend test. LOQ: limit of quantification.

a 0.01 ng/mL.

b Urinary cotinine levels above LOQ.

## DISCUSSION

Our study showed that nearly 90% of self-reported non-smoking workers had detectable urinary cotinine concentrations despite working in a factory with a smoke-free workplace policy, whereas only one in five reported awareness of SSA exposure. These findings indicate that SSA exposure outside the workplace remains highly prevalent and that most exposure may occur without conscious recognition by exposed individuals.

### Prevalence of SSA

The high prevalence of detectable cotinine observed in the present study is consistent with biomarkerbased studies^6,7^, suggesting that low-level nicotine exposure persists despite progressively strengthened smoking restrictions in public places and workplaces^4^. In a nationally representative survey of US non-smoking adults using NHANES 2013–2020 data, Wang et al.^6^ reported detectable serum cotinine concentrations in 51.2% of participants^6^. In Korea, urinary cotinine was detected in over 80% of non-smoking adults in nationally representative survey data collected between 2009 and 2018^7^. The present study extends these findings by examining urinary cotinine concentrations among workers employed at a smoke-free factory under current conditions in which HTPs are widely used in Japan.

The prevalence of detectable cotinine in the present study and the above Korean study^7^ was much higher than that reported in the US study^6^. This could be ascribed to the use of urinary cotinine measurements. Cotinine is an established biomarker of environmental tobacco smoke exposure^5^, and its relatively long elimination half-life of approximately 16–20 hours in body fluids allows cotinine measurements to reflect recent nicotine exposure over the preceding one to several days^14^. Urinary cotinine concentrations are generally several-fold higher than those in blood or saliva, which facilitates the detection of low-level nicotine exposure^15^. The Korean study excluded non-smokers who had cotinine levels comparable to smokers^7^, whereas we included workers with very high cotinine levels, likely accounting for the higher prevalence in our study than the Korean study.

### Factors associated with SSA

In the present study, detectable urinary cotinine was highly prevalent across participant subgroups, suggesting that low-level SSA exposure may be widespread even among non-smoking workers in a smoke-free workplace. In contrast, high urinary cotinine concentrations were not uniformly distributed: female sex, manual work, and former smoking were independently associated with higher odds of high urinary cotinine concentrations, whereas higher level of education was associated with lower odds. Some of these patterns are consistent with previous biomarker-based studies. In a Korean national study, cotinine-defined SHS exposure was more prevalent among adults engaged in blue-collar work^7^, and in a US national study, cotinine-defined SHS exposure was more prevalent among adults with lower level of education^6^. These differences may reflect social and environmental disparities in exposure opportunities, including residential environments, household smoking, social networks, and the ability to avoid settings where tobacco smoke or HTP aerosol is present. Given the strict factory-wide smoking ban in the present study, exposure within the workplace was unlikely. Therefore, the higher odds observed in these subgroups may reflect unmeasured SSA exposure outside the factory, such as at home, during commuting, in social settings, or in venues where smoking or HTP use is allowed. Former smokers may also be more likely than never smokers to live with or socialize with smokers, which could increase opportunities for unrecognized exposure.

### Awareness of SSA exposure

In the present study, there was a large gap between low self-reported awareness and high biomarker-based exposure of SSA, with nearly 80% of those with detectable urinary cotinine being unaware of their exposure. This finding is consistent with previous biomarker-based studies showing that many individuals with measurable nicotine exposure do not recognize their SSA exposure. In a 2018 Korean national survey, substantial discrepancies were observed between self-reported indoor SSA exposure (3.7% at home, 15.4% at public places, and 12.2% at workplaces) and urinary cotinine-based assessment (80.0%)^7^. In the US nationally representative survey, 22.0% self-reported SSA exposure, whereas 51.2% had detectable serum cotinine, with 67.6% of those with detectable serum cotinine being unaware of their exposure^6^. Nicotine exposure in daily life may occur intermittently in settings outside the workplace, such as building entrances, outdoor smoking areas, restaurants, bars, and commuting spaces, which people may not be aware of. The widespread use of HTPs in Japan, especially in younger working-age adults^8^, may partly explain this large gap. Compared with combustible cigarettes, HTPs generate aerosol rather than smoke and are often perceived as producing less visible emissions and less residual odor^9,16^, potentially making passive exposure less noticeable to bystanders while still delivering nicotine-containing emissions.

### Strength and limitations

A major strength of the present study is the use of urinary cotinine, an objective and sensitive biomarker of nicotine exposure, which enabled the detection of low-level exposure. In addition, the study was conducted among workers employed at a workplace with a strict smoke-free policy, providing insight into SSA exposure outside the workplace, which has been a major location where SSA exposure occurs.

However, several limitations should be considered. First, smoking status and secondhand smoke or aerosol exposure were self-reported and therefore may be subject to information bias and misclassification. In particular, some current smokers may have been misclassified as non-smokers because of inaccurate reporting of smoking status, potentially contributing to the high prevalence of detectable urinary cotinine. Second, although urinary cotinine is a useful biomarker of recent nicotine exposure, it cannot identify the specific timing, location, or source of exposure. Therefore, we were unable to determine whether the detected cotinine reflected exposure in the workplace, at home, during commuting, or in other daily life settings. Third, although multivariable logistic regression analyses were performed to adjust for measured background factors, residual confounding by unmeasured or imperfectly measured factors cannot be excluded. In addition, because this was a cross-sectional analysis, the observed associations between background factors and high urinary cotinine concentrations should not be interpreted as causal relationships. Rather, these findings should be regarded as exploratory evidence for identifying subgroups with higher levels of cotinine-detected nicotine exposure. Lastly, the study population was drawn from a workplace with a strict factory-wide smoking ban, which may limit generalizability of our findings to workplaces with less stringent smoking policies.

## CONCLUSIONS

Despite a complete smoke-free workplace policy, a large proportion of self-reported non-smoking workers had detectable urinary cotinine concentrations, and the majority were unaware of their SSA exposure. Biomarker-based assessment may help identify unrecognized nicotine exposure among non-smoking workers in smoke-free settings. Further studies are warranted to clarify exposure sources and to better characterize SSA exposure in the context of the increasing use of HTPs.

## Data Availability

The data supporting this research cannot be made available for privacy or other reasons but they can be available from the authors on reasonable request.
